# Dynamic Binder Exchange Improves Protein Labeling Efficiency in DNA‐PAINT up to 15‐Fold

**DOI:** 10.1002/anie.202518685

**Published:** 2026-02-03

**Authors:** Clemens Steinek, Isabelle Pachmayr, Sebastian Strauss, Monique Honsa, Jisoo Kwon, Ralf Jungmann

**Affiliations:** ^1^ Max Planck Institute of Biochemistry Am Klopferspitz 18 82152 Martinsried Germany; ^2^ Faculty of Physics and Center for Nanoscience LMU Munich Geschwister‐Scholl‐Platz 1 80539 Munich Germany

**Keywords:** DNA‐PAINT, Labeling, Single‐molecule Imaging, Spatial proteomics, Super‐resolution microscopy

## Abstract

Dynamic Binder Exchange (DyBE) enhances DNA‐PAINT (Point Accumulation for Imaging in Nanoscale Topography) super‐resolution microscopy by exploiting transient, reversible binder‐target interactions. DyBE uses DNA‐conjugated binders such as nanobodies as both targeting and docking moieties, integrating their characteristic higher off‐rates with DNA‐PAINT blinking to efficiently sample target sites. This dual‐kinetic scheme increases labeling efficiency up to 15‐fold, enabling sensitive detection of targets previously inaccessible due to limitations of high‐off‐rate binders. Using DyBE, the study reveals pre‐existing HER2 homodimers and ligand‐induced EGFR‐HER2 heterodimers at single‐protein resolution with high fidelity. DyBE expands the usable binder repertoire, advancing spatial proteomics and enabling mechanistic drug studies of receptor organization and signaling.

## Graphical abstract

Dynamic Binder Exchange (DyBE) leverages transient binder–target interactions to enhance labeling efficiency by up to 15‐fold in super‐resolution microscopy. DyBE maps nanoscale receptor tyrosine kinase (RTK) assemblies, including HER2 homodimers and EGFR–HER2 heterodimers, advancing single‐protein spatial proteomics.

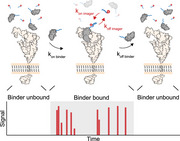

The structural integrity and functional state of cells are orchestrated by a highly dynamic and complex network of protein–protein interactions. These networks regulate fundamental cellular processes such as cell‐cell communication, signal transduction, gene regulation, and cell division.^[^ ^1^ ^]^ While biochemical assays and proximity proteomics have considerably advanced our understanding of these processes, they offer limited insight into the spatial arrangement of individual proteins and complexes within their native cellular context. Crucially, the nanoscale organization of such complexes often governs their functional states, making spatial information indispensable for a mechanistic understanding of cellular signaling and dysfunction.^[^ ^2^, ^3^ ^]^

Super‐resolution microscopy techniques are uniquely positioned to close this gap by providing the necessary spatial resolution to visualize the nanoscale organization of proteins at the single‐molecule level. DNA‐PAINT (Point Accumulation for Imaging in Nanoscale Topography)^[^ ^4^ ^]^ is a single‐molecule localization microscopy^[^ ^5^ ^]^ technique that leverages the transient binding of fluorescent DNA “imager” strands to complementary “docking” strands immobilized on, e.g., protein targets. This approach generates images with spatial resolutions better than 5 nm.^[^ ^6^, ^7^ ^]^ Recent advances in DNA‐PAINT—including improvements in imaging speed^[^ ^8^, ^9^ ^]^ spatial resolution,^[^ ^10^ ^]^ and multiplexing—have transformed our ability to generate high‐content, single‐molecule‐resolved maps of cellular processes and protein interaction networks.^[^ ^11^, ^12^ ^]^

However, accurate localization of target proteins depends not only on high spatial resolution but also on the accuracy, precision, and completeness of molecular labeling. This requires small, highly specific, and stoichiometrically defined binders that minimize steric hindrance and spatial offset, also known as linkage error. Antibodies offer high specificity and broad target availability, but their size (∼150 kDa) introduces labeling offsets of 10 to 25 nm.^[^ ^13^ ^]^ Smaller binders such as nanobodies (∼15 kDa),^[^ ^13^ ^]^ aptamers,^[^ ^14^, ^15^ ^]^ and affimers^[^ ^16^ ^]^ offer more accurate target localization and are better suited for advanced super‐resolution imaging. However, many of these small binders have moderate affinities^[^ ^17^ ^]^ and relatively high off‐rates, rendering them less suitable for classical DNA‐PAINT, where stable target anchoring is essential for high labeling efficiency. In a recent study,^[^ ^18^ ^]^ we demonstrated that high labeling efficiency is essential for accurately resolving single‐protein positions and reliably inferring stoichiometries, as incomplete labeling distorts nearest‐neighbor distributions and undermines structural interpretations. Low labeling efficiency has long been a bottleneck in expanding the labeling toolbox for high‐accuracy imaging.

To overcome this limitation, we here introduce Dynamic Binder Exchange (DyBE), a method that embraces rather than avoids transient binding of labeling probes. DyBE relies on two coupled kinetic layers that together determine target detectability and blinking kinetics: (i) the reversible interaction between binder and target, and (ii) the transient hybridization of fluorescent imager strands to the DNA docking handle on the binder. In contrast to classical DNA‐PAINT, where the binder‐target interaction is essentially static, DyBE operates in a regime where binders continuously sample the target with an on‐rate *k*_on,binder_ and an off‐rate *k*_off,binder_. The instantaneous occupancy of the target site is therefore given by

$$f_{\mathrm{occ}}=\frac{k_{\mathrm{on},\mathrm{binder}}\;c_{\mathrm{binder}}}{k_{\mathrm{on},\mathrm{binder}}\;c_{\mathrm{binder}}+k_{\mathrm{off},\mathrm{binder}}}\;.$$

Whenever a binder is present on the target, its DNA docking strand can undergo multiple imager binding events with characteristic imager on‐ and off‐rates (*k*_on,imager_, *k*_off,imager_). The observed blinking rate is therefore the convolution of (i) the binder‐target sampling frequency and dwell time and (ii) the imager binding kinetics. This dual‐kinetic architecture defines the dynamic range of DyBE: weak binders with high *k*_off,binder_ produce short sampling windows but are compensated by repeated replenishment from solution, whereas strong binders behave similarly to classical DNA‐PAINT.

DyBE thus combines the blinking kinetics of DNA‐PAINT with reversible interactions between high‐off‐rate binders and protein targets (akin to the original PAINT^[^ ^19^ ^]^ and IRIS^[^ ^20^, ^21^ ^]^ concepts). In practice, DyBE implements this dual‐kinetic scheme by maintaining both DNA‐conjugated binders and fluorescent imagers in solution, enabling continuous exchange at the target site (Figure 1a). Continuous sampling during image acquisition compensates for the low occupancy of weakly binding molecules and enhances labeling efficiency (Figure 1b).

**Figure 1:**
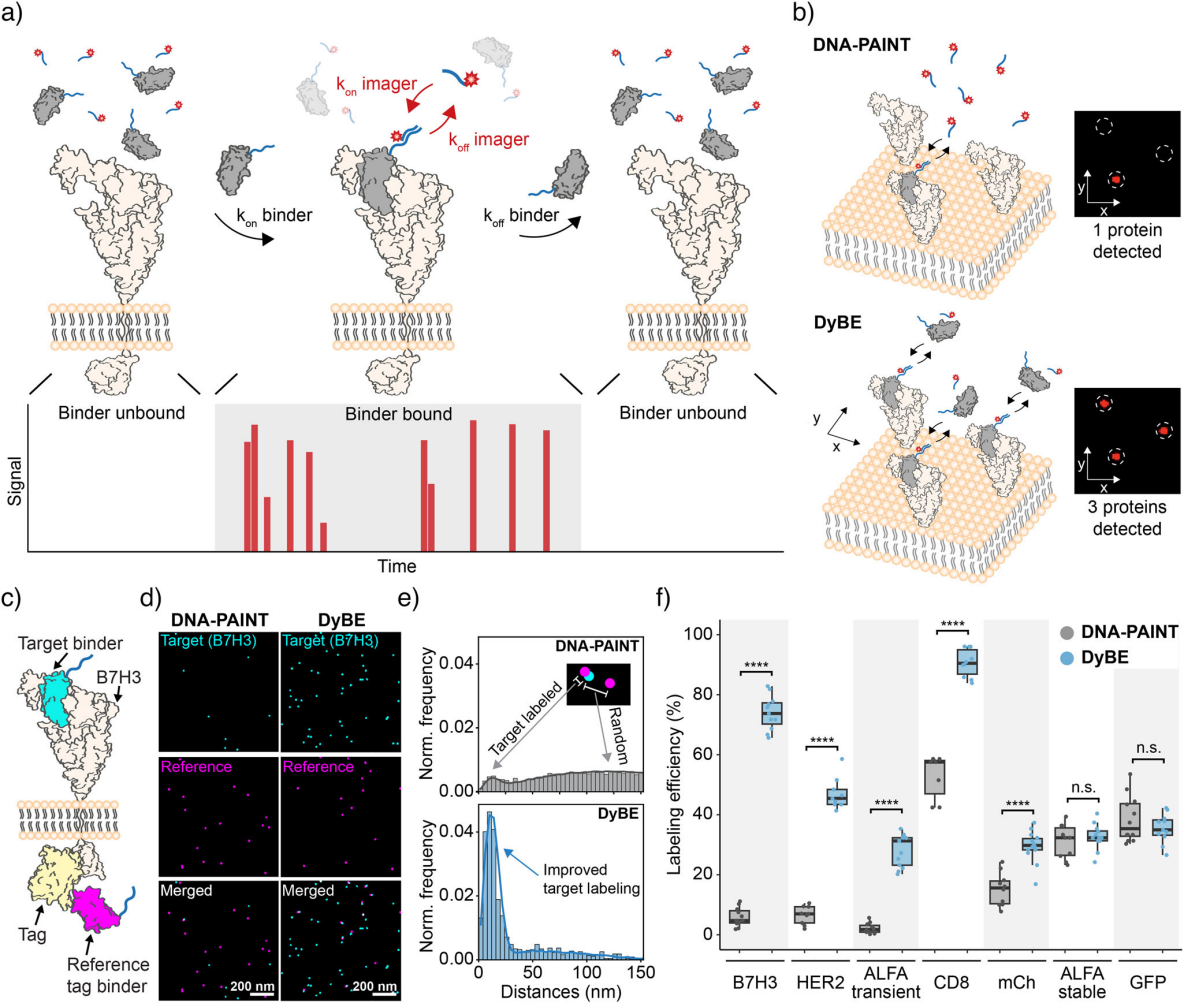
DyBE improves labeling efficiency with high‐off‐rate binders. a) DyBE concept. DNA‐conjugated labeling probes bind reversibly to their target proteins. Their positions are determined by the binding of complementary dye‐labeled imager strands. b) In classical DNA‐PAINT, the fraction of detectable protein molecules is limited by the number of binders present on the target during image acquisition. DyBE leverages repeated transient interactions between the binder and its target to enhance labeling occupancy and detection sensitivity. c) Workflow for quantifying labeling efficiency. The target protein (e.g., B7H3) is genetically fused to an intracellular reference tag (e.g., GFP or ALFA‐tag). A tag‐specific nanobody (magenta) serves as a stable reference, while the DNA‐conjugated target binder (cyan) is tested under classical DNA‐PAINT or DyBE conditions. d) Representative images of DNA‐PAINT and DyBE experiments targeting B7H3. e) Cross nearest‐neighbor distance (NND) analysis between reference and target signals. Experimental distributions (histograms) are fitted to a simulation model (solid lines) to infer labeling efficiency at the single‐protein level. f) Quantitative comparison of labeling efficiencies for different nanobodies, demonstrating significant improvements with DyBE relative to classical DNA‐PAINT. Boxplots indicate the 25^th^ and 75^th^ percentiles, with the whiskers showing the minima and maxima (5^th^ and 95^th^ percentiles), and the horizontal line showing the median. Each point represents the labeling efficiency measured in an individual cell. Significance levels were tested by two‐sample t‐tests with Bonferroni's correction for multiple testing (∗ = *p* < 0.05, ∗∗ = *p* < 0.01, ∗∗∗ = *p* < 0.001, ∗∗∗∗ = *p* < 0.0001, n.s. = nonsignificant).

We optimized DyBE conditions by testing concentration‐dependent binding saturation of different nanobodies and found that 20 nM yielded optimal signal detection with minimal nonspecific binding (Supplementary Figure 1). Additionally, using a 1.5‐fold excess of imager probes relative to classical DNA‐PAINT resulted in comparable imager strand binding frequencies (Supplementary Figure 2). To generalize these optimized experimental conditions to binders with diverse kinetic behaviors, we simulated DyBE performance across a range of *k*_off,binder_ and identified the binder and imager concentrations that yield robust target detection (Supplementary Figure 3).

Using these optimized conditions, we next quantified the achievable labeling efficiency of small binders using DyBE by employing a previously developed reference‐tagging workflow^[^ ^22^ ^]^ (Figure 1c). In brief, a reference tag was fused to a target protein, and DyBE signals were correlated with this reference tag to quantitatively measure labeling efficiency. Compared to classical DNA‐PAINT, DyBE showed a markedly higher frequency of target‐reference colocalization events and thus enhanced achievable labeling efficiency (Figure 1d, e). This improvement was particularly pronounced when using high‐off‐rate binders, where labeling efficiencies increased from 5% in DNA‐PAINT to up to 74% in DyBE under optimal conditions, a 15‐fold improvement (Figure 1f). This highlights DyBE's capacity to use previously inaccessible binders for DNA‐PAINT microscopy.

Transient binder‐target interactions could, in principle, introduce spatial offsets between binding events and lead to the hallucination of nonexistent dimers. To assess this, we used DyBE with both stable and high‐off‐rate binders to detect monomeric CD86 and found that the fraction of monomeric protein remained consistent with DNA‐PAINT results (Supplementary Figure 4). Next, we applied DyBE to detect membrane and intracellular targets, demonstrating its capability to map protein complexes across different cellular compartments (Supplementary Figure 5). We also evaluated whether DyBE can be used to detect protein targets with primary antibodies and DNA‐conjugated secondary nanobodies and observed improvements in labeling efficiency for a subset of primary antibodies compared to classical DNA‐PAINT (Supplementary Figure 6).

To explore the biological utility of DyBE, we investigated the spatial reorganization of the two receptor tyrosine kinases (RTKs)—EGFR and HER2—in response to ligand stimulation. Both receptors are clinically important RTKs whose dimerization states critically regulate oncogenic signaling.^[^ ^23^ ^]^ We used a genetically engineered Chinese hamster ovary (CHO) cell line expressing human EGFR‐mEGFP and HER2‐TagBFP2 and labeled EGFR with a high‐affinity anti‐GFP nanobody and HER2 with a direct nanobody that is characterized by a high off‐rate. We note that the TagBFP2 label on HER2 was only used to identify suitable cells for imaging and not for actual labeling of HER2. Classical DNA‐PAINT robustly visualized EGFR but failed to detect HER2 with sufficient sensitivity (Figure 2a). In contrast, DyBE resolved the spatial organization of both receptors with high fidelity (Figure 2b). Following a 5‐minute treatment with 10 nM EGF, we observed EGFR–HER2 heterodimerization and formation of higher‐order receptor clusters (Figure 2b). Notably, HER2 dimerization was detectable even in untreated cells, a feature only observable with DyBE (Figure 2b). Employing our recently developed SPINNA workflow,^[^ ^18^ ^]^ we quantitatively resolved the distribution of HER2 molecules across monomeric, homodimeric, and EGFR‐HER2 heterodimeric states in both untreated and EGF‐stimulated cells (Supplementary Figure 7). Upon EGF stimulation, the relative fraction of HER2 monomers markedly decreased, coinciding with a substantial increase in EGFR‐HER2 heterodimers, while the proportion of HER2 homodimers remained largely unchanged.

**Figure 2:**
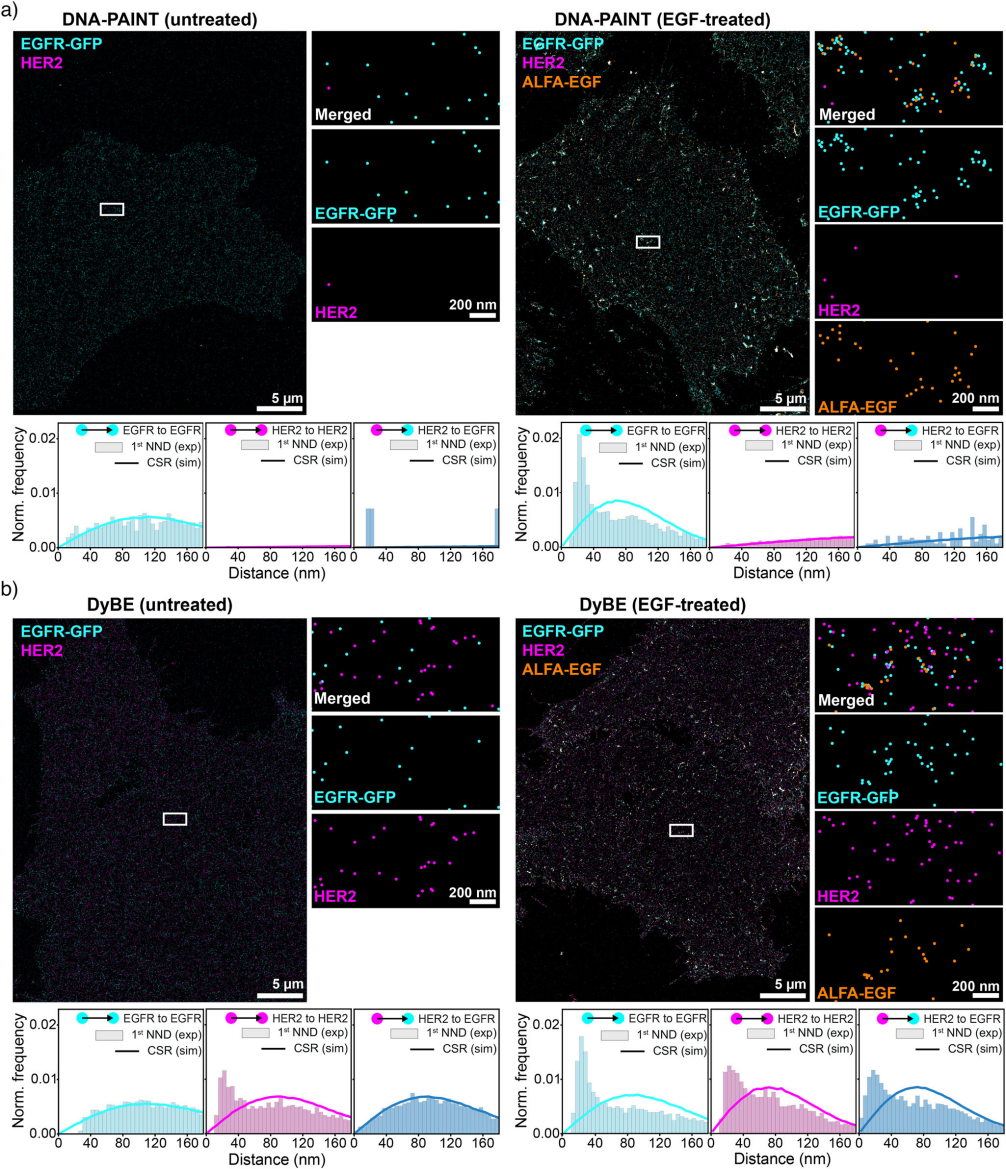
DyBE reveals shifts in RTK interactions following EGF treatment. a) Top: Representative DNA‐PAINT images of untreated and EGF‐treated cells expressing EGFR‐GFP and HER2. While EGFR‐GFP can be detected with classical DNA‐PAINT, HER2 is not effectively detected using a high‐off‐rate HER2 nanobody. Detection of ALFA‐EGF was achieved using a stable nanobody targeting the ALFA tag. Bottom: Representative analysis of first NNDs for EGFR‐EGFR, HER2‐HER2, and HER2‐EGFR dimers. The solid lines depict distributions according to complete spatial randomness (CSR). b) Top: Representative DyBE images of untreated and EGF‐treated cells expressing EGFR‐GFP and HER2. Bottom: Representative analysis of first NNDs for EGFR‐EGFR, HER2‐HER2, and HER2‐EGFR dimers. The solid lines depict distributions according to CSR. EGFR primarily remains monomeric in the absence of EGF, while HER2 forms homodimers. EGF treatment leads to the formation of EGFR homodimers and HER2‐EGFR heterodimers.

In summary, we have developed DyBE, a versatile approach to detect proteins with small, high‐off‐rate binders and probe their spatial topologies. The modular nature of DyBE makes it broadly compatible with a diverse range of small binders and protein targets. While nanobodies served as the primary model system, any DNA‐conjugatable, transient binder could, in principle, be used. This could facilitate the incorporation of binders from de novo protein design^[^ ^24^, ^25^ ^]^ or synthetic libraries^[^ ^17^ ^]^ into DNA‐PAINT imaging workflows—expanding the labeling repertoire to targets previously inaccessible by classical approaches. Its compatibility with multiplexed workflows and potentially rational binder design will make it an integral part of future spatial proteomics, systems biology, and biomedical discovery workflows.

We envision DyBE as a key component of future single‐molecule spatial proteomics workflows.^[^ ^26^ ^]^ Recent advances in DNA‐PAINT have enabled the generation of cell‐wide organellar atlases and detailed protein maps in neurons, using antibody‐based barcoding strategies.^[^ ^11^, ^12^ ^]^ The incorporation of DyBE into such frameworks could significantly enhance spatial accuracy, reduce linkage errors, and improve imaging sensitivity, allowing for the detection of rare, multimeric complexes that are elusive to conventional methods.

Improved labeling efficiency for high‐off‐rate binders not only expands the methodological landscape of super‐resolution microscopy but also opens potentially new avenues in biomedicine. Many clinically relevant epitopes, such as tumor‐specific antigens or immune checkpoint molecules, lack robust high‐affinity binders, hampering their nanoscale analysis. DyBE enables the use of transiently binding probes, including those selected from phage, yeast, or mRNA display libraries, to map the organization of these targets directly in cells. This could facilitate precision diagnostics, mechanistic studies of receptor signaling, and the spatial stratification of cellular phenotypes in tissue samples.

DyBE provides a powerful tool to dissect receptor organization and signaling at the nanoscale. Beyond revealing pre‐existing HER2 homodimers and ligand‐induced EGFR‐HER2 heterodimers, it enables a quantitative view of how receptor populations redistribute upon stimulation. Using DyBE in combination with the SPINNA analysis framework, we were able to determine how a subpopulation of HER2 shifts from a monomeric state toward heterodimerization to EGFR. Our analysis suggests that ligand engagement preferentially recruits HER2 monomers into heterodimeric signaling complexes with EGFR, while leaving pre‐existing HER2 homodimers largely unaltered. Resolving such nanoscale shifts in dimer equilibria provides critical mechanistic insight into the plasticity of HER2 signaling and its role in oncogenic activation. Importantly, many HER2‐targeted therapies—including monoclonal antibodies and small‐molecule inhibitors – act by modulating dimerization interfaces, yet current assays cannot directly visualize these complexes in their native cellular context. DyBE bridges this gap by enabling quantitative, spatially resolved profiling of receptor assemblies at single‐protein resolution in intact cells. This capability could, for example, reveal how therapeutic antibodies such as trastuzumab or pertuzumab selectively disrupt HER2 homodimerization or prevent ligand‐induced heterodimerization, and how tyrosine kinase inhibitors reshape receptor clustering. Extending DyBE to other clinically relevant receptor families promises to accelerate the rational development of next‐generation targeted therapies and optimize combination strategies based on receptor organization states.

## Conflict of Interests

The authors declare no conflict of interest.

## Supporting information



Supporting Information

Supporting Information

## Data Availability

Raw localization microscopy datasets (in hdf5 format) have been deposited at Zenodo and are publicly available as of the date of publication with this link: https://doi.org/10.5281/zenodo.17989924.

## References

[1] J. Su , Y. Song , Z. Zhu , X. Huang , J. Fan , J. Qiao , F. Mao , Signal Transduct Target Ther 2024, 9, 196, 10.1038/s41392-024-01888-z. 39107318 PMC11382761

[2] R. Nussinov , Y. Liu , W. Zhang , H. Jang , RSC Chem Biol 2023, 4, 850–864; 10.1039/D3CB00114H. 37920394 PMC10619138

[3] T. Kovacs , F. Zakany , P. Nagy , Cancers (Basel) 2022, 14. 10.3390/cancers14040944 PMC8869822 35205690

[4] R. Jungmann , C. Steinhauer , M. Scheible , A. Kuzyk , P. Tinnefeld , F. C. Simmel , Nano Lett. 2010, 10, 4756–4761, 10.1021/nl103427w. 20957983

[5] M. Lelek , M. T. Gyparaki , G. Beliu , F. Schueder , J. Griffie , S. Manley , R. Jungmann , M. Sauer , M. Lakadamyali , C. Zimmer , Nat Rev Methods Primers 2021, 1, 39, 10.1038/s43586-021-00038-x. 35663461 PMC9160414

[6] J. Schnitzbauer , M. T. Strauss , T. Schlichthaerle , F. Schueder , R. Jungmann , Nat. Protoc. 2017, 12, 1198–1228; 10.1038/nprot.2017.024. 28518172

[7] M. Dai , R. Jungmann , P. Yin , Nat. Nanotechnol. 2016, 11, 798–807, 10.1038/nnano.2016.95. 27376244 PMC5014615

[8] S. Strauss , R. Jungmann , Nat. Methods 2020, 17, 789–791; 10.1038/s41592-020-0869-x. 32601424 PMC7610413

[9] F. Schueder , J. Stein , F. Stehr , A. Auer , B. Sperl , M. T. Strauss , P. Schwille , R. Jungmann , Nat. Methods 2019, 16, 1101–1104, 10.1038/s41592-019-0584-7. 31591576

[10] S. C. M. Reinhardt , L. A. Masullo , I. Baudrexel , P. R. Steen , R. Kowalewski , A. S. Eklund , S. Strauss , E. M. Unterauer , T. Schlichthaerle , M. T. Strauss , C. Klein , R. Jungmann , Nature 2023, 617, 711–716, 10.1038/s41586-023-05925-9. 37225882 PMC10208979

[11] E. M. Unterauer , S. Shetab Boushehri , K. Jevdokimenko , L. A. Masullo , M. Ganji , S. Sograte‐Idrissi , R. Kowalewski , S. Strauss , S. C. M. Reinhardt , A. Perovic , C. Marr , F. Opazo , E. F. Fornasiero , R. Jungmann , Cell 2024, 187, 1785–1800. e16 e1716; 10.1016/j.cell.2024.02.045. 38552614

[12] F. Schueder , F. Rivera‐Molina , M. Su , Z. Marin , P. Kidd , J. E. Rothman , D. Toomre , J. Bewersdorf , Cell 2024, 187, 1769–1784.e18 e1718, 10.1016/j.cell.2024.02.033. 38552613 PMC12135969

[13] J. Ries , C. Kaplan , E. Platonova , H. Eghlidi , H. Ewers , Nat. Methods 2012, 9, 582–584, 10.1038/nmeth.1991. 22543348

[14] S. Strauss , P. C. Nickels , M. T. Strauss , V. Jimenez Sabinina , J. Ellenberg , J. D. Carter , S. Gupta , N. Janjic , R. Jungmann , Nat. Methods 2018, 15, 685–688; 10.1038/s41592-018-0105-0. 30127504 PMC6345375

[15] F. Opazo , M. Levy , M. Byrom , C. Schafer , C. Geisler , T. W. Groemer , A. D. Ellington , S. O. Rizzoli , Nat. Methods 2012, 9, 938–939, 10.1038/nmeth.2179. 23018995

[16] C. Tiede , R. Bedford , S. J. Heseltine , G. Smith , I. Wijetunga , R. Ross , D. AlQallaf , A. P. Roberts , A. Balls , A. Curd , R. E. Hughes , H. Martin , S. R. Needham , L. C. Zanetti‐Domingues , Y. Sadigh , T. P. Peacock , A. A. Tang , N. Gibson , H. Kyle , G. W. Platt , N. Ingram , T. Taylor , L. P. Coletta , I. Manfield , M. Knowles , S. Bell , F. Esteves , A. Maqbool , R. K. Prasad , M. Drinkhill , et al., Elife 2017, 6. 10.7554/eLife.24903 PMC5487212 28654419

[17] I. Zimmermann , P. Egloff , C. A. Hutter , F. M. Arnold , P. Stohler , N. Bocquet , M. N. Hug , S. Huber , M. Siegrist , L. Hetemann , J. Gera , S. Gmur , P. Spies , D. Gygax , E. R. Geertsma , R. J. Dawson , M. A. Seeger , Elife 2018, 7. 10.7554/eLife.34317 PMC5967865 29792401

[18] L. A. Masullo , R. Kowalewski , M. Honsa , L. Heinze , S. Xu , P. R. Steen , H. Grabmayr , I. Pachmayr , S. C. M. Reinhardt , A. Perovic , J. Kwon , E. P. Oxley , R. A. Dickins , M. M. C. Bastings , I. A. Parish , R. Jungmann , Nat. Commun. 2025, 16, 4202, 10.1038/s41467-025-59500-z. 40328783 PMC12056017

[19] A. Sharonov , R. M. Hochstrasser , Proc Natl Acad Sci U S A 2006, 103, 18911–18916, 10.1073/pnas.0609643104. 17142314 PMC1748151

[20] Q. Zhang , A. Miyamoto , S. Watanabe , T. Arimori , M. Sakai , M. Tomisaki , T. Kiuchi , J. Takagi , N. Watanabe , Cell Rep Methods 2022, 2, 100301; 36313806 10.1016/j.crmeth.2022.100301 PMC9606137

[21] T. Kiuchi , M. Higuchi , A. Takamura , M. Maruoka , N. Watanabe , Nat. Methods 2015, 12, 743–746, 10.1038/nmeth.3466. 26147917

[22] J. Hellmeier , S. Strauss , S. Xu , L. A. Masullo , E. M. Unterauer , R. Kowalewski , R. Jungmann , Nat. Methods 2024, 21, 1702–1707, 10.1038/s41592-024-02242-5. 38658647 PMC11399078

[23] M. A. Lemmon , J. Schlessinger , Cell 2010, 141, 1117–1134, 10.1016/j.cell.2010.06.011. 20602996 PMC2914105

[24] L. Cao , B. Coventry , I. Goreshnik , B. Huang , W. Sheffler , J. S. Park , K. M. Jude , I. Markovic , R. U. Kadam , K. H. G. Verschueren , K. Verstraete , S. T. R. Walsh , N. Bennett , A. Phal , A. Yang , L. Kozodoy , M. DeWitt , L. Picton , L. Miller , E. M. Strauch , N. D. DeBouver , A. Pires , A. K. Bera , S. Halabiya , B. Hammerson , W. Yang , S. Bernard , L. Stewart , I. A. Wilson , H. Ruohola‐Baker , et al., Nature 2022, 605, 551–560; 10.1038/s41586-022-04654-9. 35332283 PMC9117152

[25] S. J. Fleishman , T. A. Whitehead , D. C. Ekiert , C. Dreyfus , J. E. Corn , E. M. Strauch , I. A. Wilson , D. Baker , Science 2011, 332, 816–821, 10.1126/science.1202617. 21566186 PMC3164876

[26] F. Schueder , E. M. Unterauer , M. Ganji , R. Jungmann , Proteomics 2020, 20, e1900368. 33030780 10.1002/pmic.201900368

