# Supplementary material for: Dynamic Binder Exchange Improves Protein Labeling Efficiency in DNA‐PAINT up to 15‐Fold
Source: Angew Chem Int Ed Engl. 2026 Feb 3;65(11):e18685. doi: 10.1002/anie.202518685 (PMC12970519; doi:10.1002/anie.202518685)
Supplement: Supplementary file 1 — Supporting Information [file ANIE-65-e18685-s001.docx]

Supplementary Information

**Dynamic Binder Exchange Improves Protein Labeling Efficiency in DNA-PAINT up to 15-Fold**

Materials and Methods

**Materials.** Unmodified DNA oligonucleotides were purchased from MWG Eurofins and Metabion. DNA oligonucleotides modified with C3-azide and Cy3B were ordered from Metabion and MWG Eurofins. Magnesium chloride (1 M; AM9530G), sodium chloride (5 M; AM9759), ultrapure water (10977-035), Tris (1 M, pH 8; AM9855G), EDTA (0.5 M, pH 8.0; AM9260G) and 10× PBS (70011051) were purchased from Thermo Fisher Scientific. BSA (A4503-10G) was ordered from Sigma-Aldrich. Triton X-100 (6683.1) was purchased from Carl Roth. Sodium hydroxide (31627.290) was purchased from VWR. Paraformaldehyde (PFA, 15710) and glutaraldehyde (16220) were obtained from Electron Microscopy Sciences. Tween-20 (P9416-50ML), glycerol (65516-500ml), methanol (32213-2.5L), and (±)-6-hydroxy-2,5,7,8-tetra-methylchromane-2-carboxylic acid (Trolox; 238813-5G) were ordered from Sigma-Aldrich.FBS (10500-064), 1× PBS (pH 7.2; 20012-019), 0.05% trypsin–EDTA (25300- 054), Salmon Sperm DNA (15632011), OptiMEM (31985062), NH_4_Cl (012361.36), NaN_3_ (190380050), and Lipofectamine 3000 (L3000015) were purchased from Thermo Fisher Scientific. Ninety-nanometer gold nanoparticles (G-90-100) were ordered from Cytodiagnostics. Sulfo DBCO-PEG_4_-Maleimide (BP-23318) and DBCO-AF647 (CLK-1302A) were purchased from Jena Bioscience.

**Buffers.** The following buffers were used for sample preparation and imaging:

- Buffer C: 1× PBS, 1 mM EDTA, and 500 mM NaCl, pH 7.4; 0.02 % Tween; optionally supplemented with 1× Trolox
- Blocking buffer: 1× PBS, 1 mM EDTA, 0.02% Tween-20, 0.05 % NaN_3_, 2 % BSA, 0.05 mg/mL sheared salmon sperm DNA
- Quenching buffer: 0.2 M NH_4_Cl in 1xPBS

**Trolox.** A 100× Trolox stock solution was prepared by dissolving 100 mg of Trolox in 430 µl of 100 % methanol, 345 µl of 1 M NaOH, and 3.2 mL of water.

**Microscope setup.** Fluorescence imaging was carried out on an inverted microscope (Nikon Instruments, Eclipse Ti2) with a Perfect Focus System, applying an objective-type TIRF configuration equipped with an oil-immersion objective (Nikon Instruments, Apo SR TIRF×100, NA 1.49, Oil). A 560-nm laser (MPB Communications, 1 W) was used for excitation and coupled into the microscope via a Nikon manual TIRF module. The laser beam was passed through a cleanup filter (Chroma Technology, ZET561/10) and coupled into the microscope objective using a beam splitter (Chroma Technology, ZT561rdc). Fluorescence was spectrally filtered with an emission filter (Chroma Technology, ET600/50m and ET575lp) and imaged on an sCMOS camera (Hamamatsu Fusion BT) without further magnification, resulting in an effective pixel size of 130 nm after 2×2 binning. TIR illumination was used for all measurements. The central 1152×1152 pixels (576×576 after binning) of the camera were used as the region of interest. The scan mode of the camera was set to “ultra quiet scan” (readout noise = 0.7 e^-^ r.m.s., 80 μs readout time per line). Raw microscopy data was acquired using μManager (Version 2.0.1)^[1]^.

**Nanobody-DNA conjugation via C-terminal cysteine.** A detailed description of the DNA-nanobody conjugation can be found in Strauss et al.^[2]^. Briefly, nanobodies containing a C-terminal cysteine were reacted with a 10-fold molar excess of bifunctional maleimide-PEG_4_-DBCO linker (Sigma-Aldrich, cat: 760668) for 2-3 h on ice. Unreacted linker was removed using Amicon centrifugal filters (10,000 MWCO), and the resulting DBCO-labeled nanobody was conjugated to the corresponding 5'-azide-modified docking strand using DBCO-azide click chemistry (Supplementary Table 1).

**Nanobody-DNA conjugation via Sortase.** Nanobodies carrying a C-terminal LPETG-HHHHHH tag were incubated with Sortase 7M^[3]^ and GGG-DNA at a 0.25-fold and 3-fold molar ratio, respectively, in 50 mM Tris-HCl, 150 mM NaCl, pH 7.5, supplemented with 10 mM CaCl₂. Azide-DNA was reacted overnight at 4 °C with a DBCO-PEG4-Gly-Gly-Gly linker (Vectorlabs, cat. no. CCT-1552) to obtain GGG-DNA, which was then added to the sortase reaction. The reactions were then carried out at 25 °C for 1 h.

**Nanobody purification.** Unconjugated nanobody and free 5'-azide-modified DNA were separated from DNA-conjugated nanobodies by anion exchange chromatography using an ÄKTA Pure liquid chromatography system equipped with a Resource Q 1 mL column (Cytiva, cat:17117701). The concentrated DNA-conjugated nanobody was adjusted to a concentration of 5-10 µM in 1× PBS, containing 50% glycerol and 0.02% NaN₃ and stored at -20 °C for 1-6 months or at -80 °C for >6 months.

**Expression and purification of ALFA-EGF.** A detailed description of the ALFA-EGF production can be found in Honsa et al.^[4]^. Briefly, GB1-TEV-ALFA-GSGS-EGF was expressed and extracted from SHuffle® T7 Express Competent E. coli^[5]^. For GST Affinity Purification, the clarified lysate was loaded onto a Glutathione Sepharose 4B column equilibrated with 50 mM Tris-HCl (pH 7.5) and 200 mM NaCl. After washing the column with 10 column volumes of binding buffer, ALFA-EGF was eluted by cleavage with His-tagged TEV protease (1 mg/mL in 50 mM Tris-HCl, 200 mM NaCl) during an overnight incubation at 4 °C, which removed the GST-GSGS-GB1 tag. The eluted ALFA-EGF was further purified using a Superdex 30 Increase GL 10/300 column equilibrated with 50 mM Tris-HCl (pH 7.5) and 150 mM NaCl. Fractions containing purified ALFA-EGF were pooled, and protein samples were analyzed by SDS-PAGE using 4–22% gradient gels under non-reducing conditions.

**Cloning.** For transient transfection, gBlocks containing the genes of interest were ordered from IDT and cloned into a pcDNA3.1(+) mammalian expression vector (Thermo Fisher Scientific, cat. V79020) using Gibson assembly (NEB, E2611S). To test tag-binders, TagBFP2, mCherry, and mEGFP were cloned as C-terminal tags onto mouse CD86, which carried an N-terminal ALFA-tag. For labeling efficiency measurements, the genes of interest were fused to a C-terminal ALFA-mEGFP tag. To generate doxycycline-inducible stable cell lines, donor plasmids (pUT7-EGFR-GFP and pUT7-HER2-TagBFP2) were derived from PB-T-PAF^[6]^. These vectors contained the minimal UCOE CBX3 sequence^[7]^, and the genes of interest were cloned into them.

**Cell culture.** Wild-type CHO cells were cultured at 37 °C and 5% CO_2_ in Ham's F-12K medium (Gibco, no. 21127022) supplemented with 10% FBS (Gibco, no. 11573397).

Doxycycline-inducible CHO cell lines were maintained in Ham's F-12K medium (Gibco, no. 21127022) supplemented with 10% FBS (Gibco, no. 11573397) and 1 μg/mL puromycin (Thermo Fisher Scientific, no. A1113803). U2OS Nup96-Halo cells^[8]^ were maintained in McCoy's 5A (Modified) Medium (Thermo Fisher Scientific, no. 16600082) supplemented with 10% FBS (Gibco, no. 11573397). Cells were passaged every 2-3 days using trypsin-EDTA (Gibco, no. 25300096).

**PiggyBac transposition.** 500,000 CHO cells were transfected in a six-well plate with 0.625 μg pUT7-EGFR-GFP, 0.625 μg pUT7-HER2-TagBFP2, 0.625 μg PiggyBac transposase vector^[9]^, and 0.625 μg reverse tetracycline transactivator plasmid^[6]^ using Lipofectamine3000 (Thermo Fisher Scientific, no. L3000008) according to the manufacturer's instructions. After 48 h, cells were plated at 40% confluency in a p100 cell culture dish, and 1 μg/mL puromycin (Thermo Fisher Scientific, no. A1113803) was added for selection. Cells were passaged at least twice under antibiotic selection pressure. Subsequently, 0.5 μg/mL doxycycline was added for 48 h, and positive cells were enriched by fluorescence-activated cell sorting (FACS) using a BD FACSAria II.

**Transient transfection and sample preparation.** 10,000 cm^-2^ wild-type CHO cells were seeded on eight-well high glass-bottom chambers (Ibidi, no. 80807). The next day, cells were transfected with 250 ng of plasmid per well using Lipofectamine 3000 (Thermo Fisher Scientific, no. L3000008), according to the manufacturer’s instructions. After 16-24 h, cells were fixed with pre-warmed 4% methanol-free PFA for 15 min and washed three times with 1x PBS. The sample was then permeabilized with 0.1% TritonX-100 in 1x PBS for 5 min at room temperature, washed three times with 1x PBS and blocked overnight at 4 °C with blocking buffer.

**Titration imaging.** Transiently transfected cells were fixed and blocked as described above. Samples were washed three times with 1× PBS. The samples were incubated with a 1:1 mixture of 90 nm gold nanoparticles in 1× PBS for 6 min at room temperature, followed by three washes with 1× PBS. Transfected cells were selected at the microscope for low and homogenous GFP expression. Before imaging, Buffer C was freshly supplemented with 1× Trolox and 1 nM R3-Cy3b imager, and added to the respective well. After 5 min, 5,000 frames per field of view were acquired in TIRF mode with an exposure time of 100 ms per frame. A laser power of 30 mW (560 nm, measured after the objective) was used, corresponding to a power density of ~150 W/cm². After the measurement, the well was washed three times with 1× PBS. Buffer C, freshly supplemented with 1× Trolox, 1 nM nanobody, and 1 nM R3-Cy3b imager, was added to the well. After min of incubation, the same fields of view were imaged at 560 nm using the same imaging conditions as in the first round. This sequence was repeated with increasing nanobody concentrations (10, 20, 50, 100 nM) in the imaging buffer until the maximum concentration was reached.

Untransfected CHO cells were used as negative controls and were fixed, permeabilized, and blocked as previously described. Cells were randomly selected for imaging and imaged with Buffer C supplemented with Trolox and increasing concentrations of nanobody, following the same procedure as for the transfected cells.

**Single-molecule localization analysis.** Raw fluorescence data were reconstructed using the Picasso software package^[10]^ (the latest version is available at <https://github.com/jungmannlab/picasso>). Drift correction was performed with AIM^[11]^. Localizations were filtered by the width of single emitter images (“sx” and “sy”, values between 0.6 and 1.2 were kept) and ellipticity (values below 0.15 were kept) to remove crosstalk. Multi-channel datasets were aligned using redundant cross-correlation^[12]^, followed by using gold particles as fiducials.

**Titration imaging analysis.** The same regions in a cell were selected for each imaging condition from the localized, undrifted, filtered, and aligned data. The raw number of localizations/μm^2^ was extracted for each condition in each cell (Supplementary Fig. 1b). Binding curves were generated by subtracting the average background signal of untransfected cells from the average signal of transfected cells for each condition. The signals were then normalized by dividing them by the value from the condition with the maximum number of localizations. Binding curves were fitted using a three-parameter log-logistic function.

**Labeling efficiency imaging of DyBE.** Transiently transfected CHO cells were fixed and blocked as described above. The samples were incubated with a 1:1 mixture of 90 nm gold nanoparticles in 1× PBS for 6 min at room temperature, followed by three washes with 1× PBS. Transfected cells were selected at the microscope for low and homogenous GFP expression. The sample was incubated with 5 nM ALFA-Nb-7xR4 (reference) in blocking buffer for 30 min, followed by three washes with 1× PBS. The sample was washed once and then incubated with buffer C containing 1x Trolox and 1 nM R4-Cy3b imager. The reference channel was imaged for 15,000 frames with an integration time of 100 ms per frame. The well was then washed six times with 400 µL of 1x PBS. Buffer C, supplemented with 1× Trolox, 20 nM target nanobody, and 1.5 nM imager, was then added to the well. The target channel was imaged for 15,000 frames with an integration time of 100 ms per frame.

**Labeling efficiency imaging of DNA-PAINT.** Transiently transfected CHO cells were fixed and blocked as described above. Samples were incubated with 5 nM ALFA-Nb-7xR4 (reference) and 50 nM target nanobody (target) in blocking buffer for 1 h at room temperature. The samples were then washed three times with 1× PBS, each wash lasting three min. The sample was postfixed with 4% methanol-free PFA and 0.1% glutaraldehyde in 1x PBS for 10 min and washed again three times with 1x PBS. Then, the samples were quenched with 0.2 M NH_4_Cl in 1x PBS for 5 min at room temperature and washed three times with 1x PBS. A 1:1 dilution of 90 nm gold nanoparticles in 1× PBS was added to the samples at room temperature for 6 min, followed by three washes with 1× PBS. Transfected cells were selected at the microscope for low and homogenous GFP expression. Buffer C containing 1× Trolox, and 1 nM R4-Cy3b imager was added to the sample, and the reference was imaged for 15,000 frames with an integration time of 100 ms per frame. After image acquisition, the well was washed 6x with 400 μL 1x PBS. To image the target nanobody, Buffer C containing 1× Trolox and 1 nM of the appropriate imager was added to the sample, and 15,000 frames were acquired with a 100 ms integration time per frame.

**Cluster analysis.** Drift-corrected, filtered and aligned localizations were processed using the Picasso SMLM clustering algorithm^[10]^ (the latest version is available at <https://github.com/jungmannlab/picasso>). Circular clusters of localizations centered around local maxima were identified and grouped (assigning a unique identification number). Subsequently, the centers of the localization groups were calculated as weighted means, using the squared inverse of localization precisions as weights.

**Labeling efficiency analysis.** Labeling efficiency was determined as described previously^[13]^. Briefly, cluster analysis was performed on the reference and the target data sets to obtain the centers of the localization groups. The cross-nearest-neighbor distances from target to reference were calculated and compared to those of simulated point-patterns. For this, simulations of variable proportions of reference monomers, target monomers and reference-target “heterodimers” were performed. The most likely proportions of populations of reference monomers (p_Ref_) and reference-target-heterodimers (p_Ref+Target_) were obtained through SPINNA^[14]^. The labeling efficiency is then calculated as LE (%) = (p_Ref+Target_/(p_Ref_+ p_Ref+Target_))×100. To quantify the proportion of monomeric CD86, cross-nearest-neighbor distances of ALFA-CD86-GFP were calculated for each channel and compared to distances from simulated point patterns. The most likely proportions of populations of monomers and homodimers were obtained through SPINNA^[14]^.

**DyBE or DNA-PAINT using primary antibodies and secondary nanobodies.** Cells were fixed and blocked as described above. Samples were washed three times with 1× PBS. A 1:1 dilution of 90 nm gold nanoparticles in 1× PBS was added to the samples at room temperature for 6 min, followed by three washes with 1× PBS. The sample was then treated as follows, depending on whether DyBE or DNA-PAINT was used:

For DyBE, 25 nM primary antibody and 62.5 nM secondary nanobody were pre-incubated in 30 μL of Blocking buffer for 1 h at 4 °C, with concentrations calculated for a final volume of 500 μL. After pre-incubation, imaging buffer supplemented with 1 nM imager strand and 1× Trolox was added to bring the final volume to 500 μL. The sample was first washed with 200 μL of the imaging buffer, followed by the addition of 300 μL. After image acquisition, the sample was washed with 1x PBS until no blinking was observed. The sample was then washed with 200 μL of buffer C containing 5 nM reference nanobody, 1 nM imager, and 1× Trolox, followed by the addition of 300 μL of the same solution for imaging. All imaging was performed with a 100 ms exposure time over 20,000 frames, using 30 mW laser power measured after the objective, corresponding to a power density of 150 W/cm².

For DNA-PAINT, 25 nM primary antibody and 62.5 nM secondary nanobody were pre-incubated in 30 μL of Blocking buffer for 1 h at 4 °C, with concentrations calculated for a final volume of 200 μL. The pre-incubated solution was diluted with blocking buffer to a final volume of 200 μL and applied to the sample for 1 h at room temperature. After incubation, the sample was washed three times with 1× PBS, followed by a 5 min incubation with buffer C at room temperature. The sample was then post-fixed with 4% methanol-free PFA and 0.1% glutaraldehyde in 1x PBS in 1× PBS for 10 min at room temperature, washed three times with 1x PBS and quenched with 0.2M NH_4_Cl in 1x PBS for 5 min at room temperature. The sample was washed three times with 1× PBS before imaging in buffer C containing 1 nM of the appropriate imager and 1× Trolox. All imaging was performed with a 100 ms exposure time over 20,000 frames, using 30 mW laser power measured after the objective, corresponding to a power density of 150 W/cm².

**EGFR-mEGFP HER2-TagBFP2 imaging.** Stable CHO cells were treated with 0.5 µg / mL doxycycline for 72 h. 10,000 cm^-2^ cells were then seeded on eight-well high glass-bottom chambers (Ibidi, no. 80807). The next day, cells were washed three times with serum-free Ham's F-12K medium (Gibco, no. 21127022) medium and starved for 24 h in serum-free Ham's F-12K medium (Gibco, no. 21127022). On the next day, cells in the ‘treated’ condition were incubated for 5 min with 10 nM ALFA-EGF in serum-free DMEM. ‘Untreated’ cells were not treated with ALFA-EGF. Cells were fixed with pre-warmed 4% methanol-free PFA (Thermofisher, no. 043368.9M) in 1× PBS for 15 min. After washing three times with 1× PBS, cells were permeabilized 0.1 % TritonX-100 (Sigma Aldrich, no. 93443) in 1× PBS for 5 min. Cells were washed three times with 1× PBS and incubated overnight at 4 °C in blocking buffer. The next day, samples were incubated with 90 nm gold nanoparticles (Absource, no. G-90-100), diluted 1:1 in 1× PBS, for 5 min at room temperature. Cells were washed three times with 1× PBS. Buffer C, freshly supplemented with 1× Trolox, 20 nM nanobody, and 1.5 nM R3-Cy3b imager, was added to the well. Cells were selected at the microscope based on low and homogeneous expression of GFP and TagBFP2. Imaging was performed over 20,000 frames with 30 mW laser power and an integration time of 100 ms per frame.

**Nup96-Halo imaging.** 15,000 cm^-2^ stable U2OS Nup96-Halo cells were seeded on eight-well high glass-bottom chambers (Ibidi, no. 80807). Cells were fixed with pre-warmed 2.4% methanol-free PFA (Thermofisher, no. 043368.9M) in 1× PBS for 30 min. After washing three times with 1× PBS, cells were permeabilized with 0.25 % TritonX-100 (Sigma Aldrich, no. 93443) in 1× PBS for 90 min. Cells were washed three times with 1× PBS and incubated overnight at 4 °C in blocking buffer. The next day, samples were incubated with 90 nm gold nanoparticles (Absource, no. G-90-100), diluted 1:1 in 1× PBS, for 5 min at room temperature. Cells were washed three times with 1× PBS. Buffer C, freshly supplemented with 1× Trolox, 20 nM anti-Halo nanobody, and 75 pM R3-Cy3b imager, was added to the well. Highly Inclined and Laminated Optical (HILO) imaging was performed over 50,000 frames with 18 mW laser power and an integration time of 100 ms per frame.

**DyBE and DNA-PAINT *in silico.*** We implemented a stochastic simulation framework to model the binder and imager kinetics of DyBE and compare them to DNA-PAINT simulations. Each target site is represented as a two-state system, with binding and unbinding transitions simulated using an event-driven exponential waiting-time process. In the DNA-PAINT model, the probability of transition from unbound to bound is governed by an effective association rate $k_{\mathrm{on}}\cdot c_{\mathrm{imager}}$, and the transition from bound to unbound is governed by $k_{\mathrm{off}}$, producing continuous timelines of ON (bound) and OFF (unbound) states.

In DyBE, imager binding occurs only during periods in which a transient binder is bound. Binder occupancy is modeled as a second two-state system with association and dissociation rates $k_{\mathrm{on},\mathrm{binder}}\cdot c_{\mathrm{binder}}$ and $k_{\mathrm{off},\mathrm{binder}}$. To account for scavenging of imagers by free binder in solution, the free imager concentration is computed from the quadratic mass–balance equation describing the reaction

$$I+A\rightleftharpoons IA$$

with dissociation constant $K_{D}$. Denoting total imager and binder concentrations by $I_{t}$ and $A_{t}$, and the complex concentration by $x=[IA]$, mass conservation gives

$$(I_{t}-x)(A_{t}-x)=K_{D}x.$$

Rearranging yields the quadratic equation

$$x^{2}-x(I_{t}+A_{t}+K_{D})+I_{t}A_{t}=0.$$

Solving for $x$(taking the physically relevant root) provides the free imager concentration $I_{\mathrm{free}}=I_{t}-x$, and the free fraction

$$f=\frac{I_{\mathrm{free}}}{I_{t}}.$$

The **effective** imager association rate in DyBE is then scaled by the free fraction:

$$k_{\mathrm{on},\mathrm{eff}}=k_{\mathrm{on},\mathrm{DNA}}\cdot I_{t}\cdot f.$$

Continuous-time traces are discretized into frames of duration $\Delta t$; a frame is marked ON if any part contains a bound imager. Sequential ON frames separated by $\leq2$ OFF frames are merged into single binding events. Simulations are performed for a range of binder kinetic parameters ($k_{\mathrm{on},\mathrm{binder}}={10}^{6}\text{ }M^{-1}s^{-1}$; $k_{\mathrm{off},\mathrm{binder}}={10}^{-6}\text{–}{10}^{-1}\text{ }s^{-1}$), binder concentrations (0.1, 1, 2, 5, 10, 20, 50, 100 nM), and imager concentrations (0.1, 0.5, 1, 1.5, 2 nM) for 10,000 frames at 100 ms per frame. For DNA-PAINT simulations, an imager concentration of 1 nM was used. The imager kinetics are kept constant in all simulations ($k_{\text{on}}$ = 44.8 * 10^6^ M^-1^ s^-1^. $k_{\text{off}}$ = 2.236  s^-1^). For each parameter set, the framework computes the mean and standard deviation of binding events per site across 2,000 simulated targets with a density of 20 targets per $\mu\text{m}^{2}$ and a DNA-PAINT reference trace.

**Supplementary Figures**

**Supplementary Figure 1 | Titration of binders. a** Representative images of the titration experiment. Cells were incubated with increasing concentrations of binders (0, 1, 10, 20, 50, 100 nM). Untransfected cells were used to measure unspecific binding. Scale bars: 4 µm. **b** Effect of nanobody concentration on number of localizations in DyBE. The raw numbers of localizations per µm^2^ over the course of image acquisition are shown for each cell. Lines depict measurements in the same cell. Untransfected cells are shown in gray. **c** Titration curves. Concentrations at which 50% of the maximum signal is recovered are indicated. A binder concentration of 20 nM was selected for subsequent experiments.

**Supplementary Figure 2 | Optimization of imager concentration for DyBE. a** Representative images of DyBE experiments. Localizations within the same cell were measured using 20 nM nanobody in solution with increasing imager concentrations (1, 1.5, and 2 nM). **b** Comparison of binding events in DNA-PAINT and DyBE. The number of binding events per 10,000 frames is indicated. For DNA-PAINT, an imager concentration of 1 nM was used. The dotted lines indicate the mean number of binding events for DNA-PAINT (orange) and DyBE (1 nM (light blue), 1.5 nM (medium blue), and 2 nM (dark blue)).

**Supplementary Figure 3 | Simulated DyBE outcomes for binders with different k_off,binder_.** Each graph represents a simulation with a fixed on-rate ($k_{\mathrm{on},\mathrm{binder}}={10}^{6}\text{ }M^{-1}s^{-1}$) and different off-rates ($k_{\mathrm{off},\mathrm{binder}}={10}^{-6}\text{–}{10}^{-1}\text{ }s^{-1}$). The x-axis depicts binder concentrations (0.1, 1, 2, 5, 10, 20, 50, 100 nM), and the y-axis shows the average number of detected binding events per site. Each line corresponds to a different imager concentration (0.1, 0.5, 1, 1.5, 2 nM), with error bars indicating the standard deviation for each point. The dotted line indicates the mean number of binding events per site from the corresponding DNA-PAINT simulation using 1 nM imager, and the shaded green area represents the standard deviation. DyBE values falling within this shaded region reflect performance equivalent to DNA-PAINT, where each position contains a fixed binder.

**Supplementary Figure 4 | Detection of monomeric CD86 with DyBE using stable or high off-rate binders. a** Schematic of the approach. CD86 is genetically fused to an extracellular ALFA-tag and an intracellular GFP-tag and is detected with DNA-PAINT using stable ALFA- (magenta) and GFP-nanobodies (cyan), or with DyBE using either stable or high off-rate ALFA- and GFP-nanobodies. NNDs between detected protein positions are then analyzed in the separate channels. **b** Quantification of the monomeric fraction in DNA-PAINT and DyBE experiments. Statistical significance was assessed by pairwise comparisons using the Wilcoxon rank-sum test with Bonferroni correction for multiple testing (∗ = p < 0.05, ∗∗ = p < 0.01, ∗∗∗ = p < 0.001). Data for this figure are derived from Figure 1F.

**Supplementary Figure 5 | Visualization of membrane-bound and intracellular targets with DyBE. a** Left: DyBE detects HER2 dimers at the membrane. Right: Representative images of HER2-tagFP at the membrane are shown, with two zoom-ins highlighting individual HER2 dimers. Line graphs depict the intensity along the dotted lines in the zoom-ins. **b** Left: sub-5 nm localization of nuclear pore complexes (NPCs) using DyBE. NPCs were labeled with NUP96-Halo via a Halo-nanobody in DyBE experiments. Right: Representative images of NPCs labeled with NUP96-Halo are shown, including two zoom-ins highlighting individual NPCs with single Nup96 copies, and side views showing the two rings.

**Supplementary Figure 6| DyBE using primary and secondary binders improves labeling efficiency compared to DNA-PAINT for selected targets. a** Primary antibodies preincubated with DNA-conjugated secondary nanobodies reversibly bind to the target protein during image acquisition. **b** Primary antibodies against three targets (CD63, CD9, EGFR) were tested in transiently transfected cells. Boxplots indicate the 25^th^ and 75^th^ percentiles, with the whiskers showing the minima and maxima (5^th^ and 95^th^ percentiles), and the horizontal line showing the median. Each point represents the labeling efficiency measured in an individual cell.

**Supplementary Figure 7| Quantitative analysis of HER2 homo- and heterodimerization with DyBE. a** Top: NND analysis of HER2-HER2 distances in a representative untreated cell (histogram) and the corresponding SPINNA model fit of HER2 monomers and HER2 dimers (line). Bottom: NND analysis of HER2-EGFR distances in a representative untreated cell (histogram) and the corresponding SPINNA model fit of HER2-EGFR heterodimers (line). **b** Top: NND analysis of HER2-HER2 distances in a representative EGF-treated cell (histogram) and the corresponding SPINNA model fit of HER2 monomers and HER2 homodimers (line). Bottom: NND analysis of HER2-EGFR distances in a representative EGF-treated cell (histogram) and the corresponding SPINNA model fit of HER2-EGFR heterodimers (line). **c** Results of a SPINNA fit, taking into account HER2 in monomers, homodimers, and heterodimers. Each data point represents an individual cell. Height of the bar and error bars represent mean and standard deviation, respectively. Significance was tested using a Two-way ANOVA, with multiple comparisons (∗ = p < 0.05, ∗∗ = p < 0.01, ∗∗∗ = p < 0.001, ∗∗∗∗ = p < 0.0001, n.s. = non-significant).

**Supplementary References**

[1] A. D. Edelstein, M. A. Tsuchida, N. Amodaj, H. Pinkard, R. D. Vale, N. Stuurman, *J Biol Methods* **2014**, *1*.

[2] S. Strauss, R. Jungmann, *Nat Methods* **2020**, *17*, 789–791.

[3] H. J. Jeong, G. C. Abhiraman, C. M. Story, J. R. Ingram, S. K. Dougan, *PLoS One* **2017**, *12*, e0189068.

[4] M. Honsa, I. Pachmayr, L. Heinze, L. Bas, L. A. Masullo, J. Kwon, A. Perovic, B. Schulman, R. Jungmann, *Small Methods* **2025**, *9*, e2401799.

[5] J. Lobstein, C. A. Emrich, C. Jeans, M. Faulkner, P. Riggs, M. Berkmen, *Microb Cell Fact* **2012**, *11*, 56.

[6] Z. Li, I. P. Michael, D. Zhou, A. Nagy, J. M. Rini, *Proc Natl Acad Sci U S A* **2013**, *110*, 5004–5009.

[7] U. Muller-Kuller, M. Ackermann, S. Kolodziej, C. Brendel, J. Fritsch, N. Lachmann, H. Kunkel, J. Lausen, A. Schambach, T. Moritz, M. Grez, *Nucleic Acids Res* **2015**, *43*, 1577–1592.

[8] J. V. Thevathasan, M. Kahnwald, K. Cieslinski, P. Hoess, S. K. Peneti, M. Reitberger, D. Heid, K. C. Kasuba, S. J. Hoerner, Y. Li, Y. L. Wu, M. Mund, U. Matti, P. M. Pereira, R. Henriques, B. Nijmeijer, M. Kueblbeck, V. J. Sabinina, J. Ellenberg, J. Ries, *Nat Methods* **2019**, *16*, 1045–1053.

[9] K. Yusa, L. Zhou, M. A. Li, A. Bradley, N. L. Craig, *Proc Natl Acad Sci U S A* **2011**, *108*, 1531–1536.

[10] J. Schnitzbauer, M. T. Strauss, T. Schlichthaerle, F. Schueder, R. Jungmann, *Nat Protoc* **2017**, *12*, 1198–1228.

[11] H. Ma, M. Chen, P. Nguyen, Y. Liu, *Sci Adv* **2024**, *10*, eadm7765.

[12] Y. Wang, J. Schnitzbauer, Z. Hu, X. Li, Y. Cheng, Z. L. Huang, B. Huang, *Opt Express* **2014**, *22*, 15982–15991.

[13] J. Hellmeier, S. Strauss, S. Xu, L. A. Masullo, E. M. Unterauer, R. Kowalewski, R. Jungmann, *Nat Methods* **2024**, *21*, 1702–1707.

[14] L. A. Masullo, R. Kowalewski, M. Honsa, L. Heinze, S. Xu, P. R. Steen, H. Grabmayr, I. Pachmayr, S. C. M. Reinhardt, A. Perovic, J. Kwon, E. P. Oxley, R. A. Dickins, M. M. C. Bastings, I. A. Parish, R. Jungmann, *Nat Commun* **2025**, *16*, 4202.
