# Supplementary figures and images for: Dynamic Binder Exchange Improves Protein Labeling Efficiency in DNA‐PAINT up to 15‐Fold
Source: Angew Chem Int Ed Engl. 2026 Feb 3;65(11):e18685. doi: 10.1002/anie.202518685 (PMC12970519; doi:10.1002/anie.202518685)

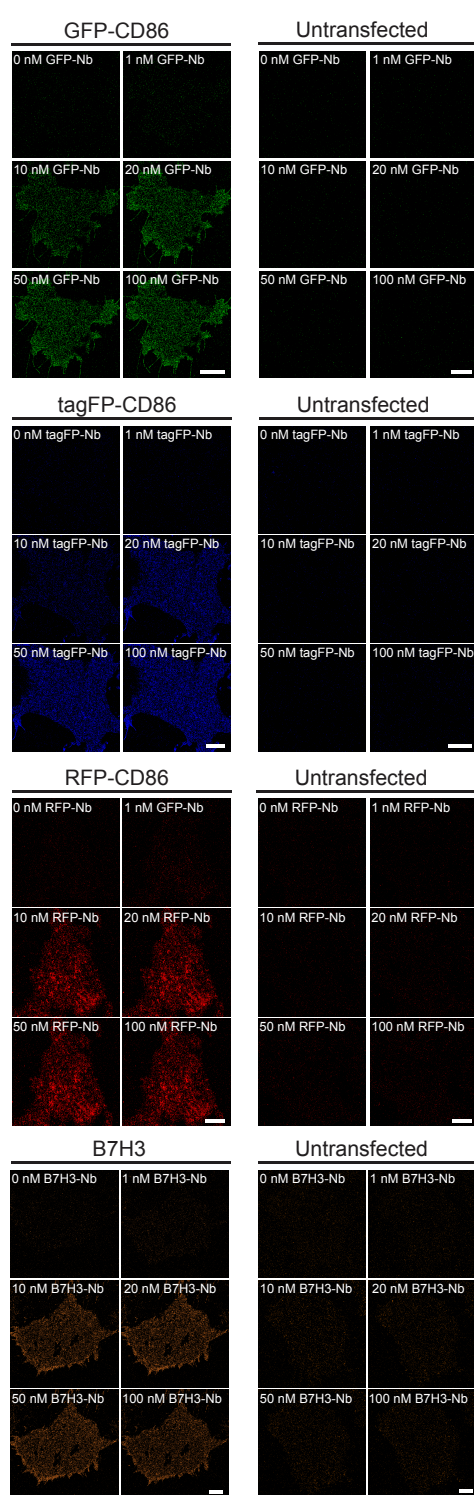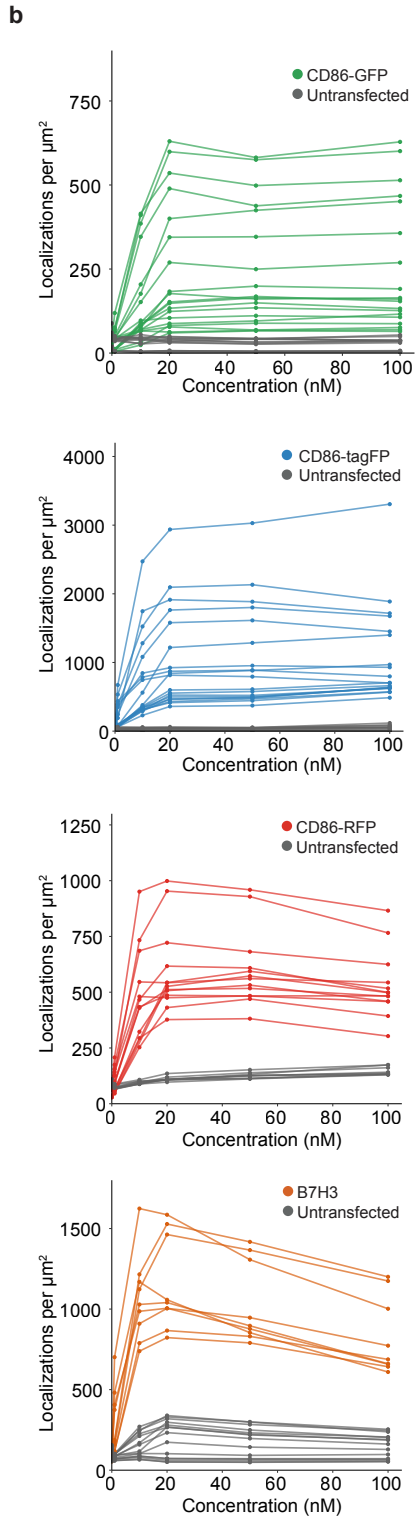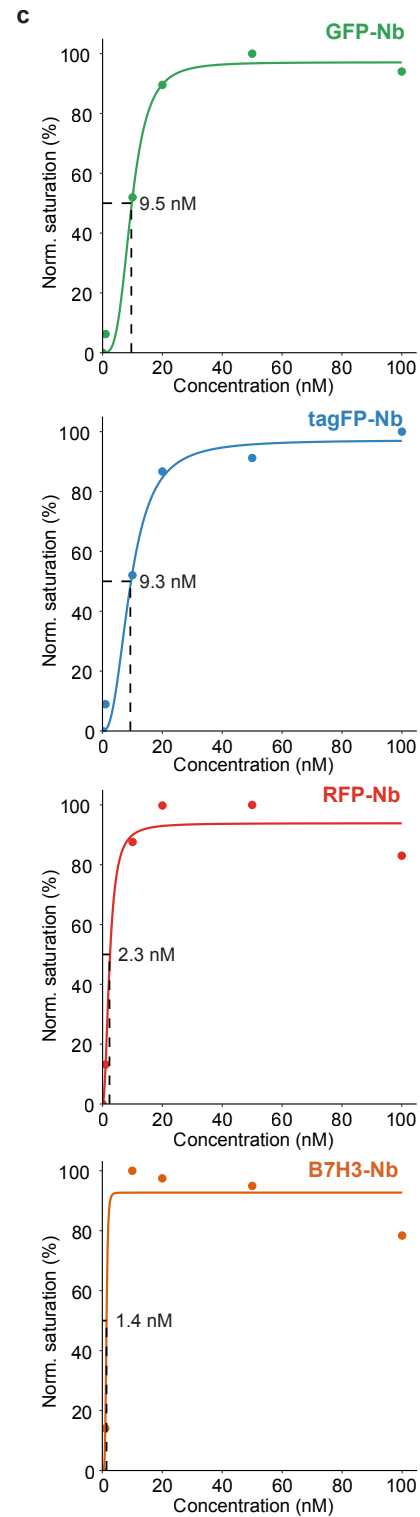

Supplement: Supplementary file 2 — Supporting Information [file ANIE-65-e18685-s002.zip › anie71221-sup-0002-Figures/anie71221-sup-0002-FigureS1.pdf]

**a**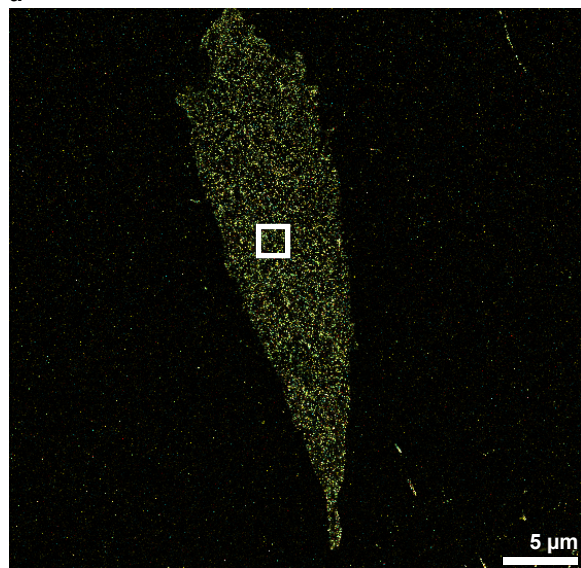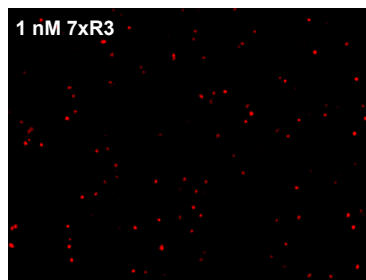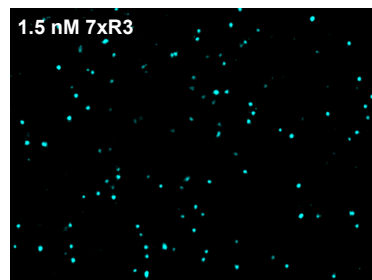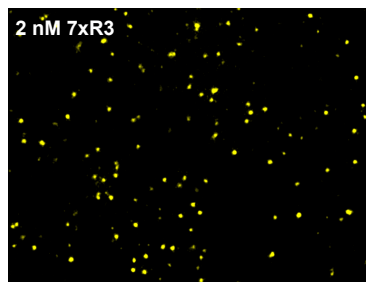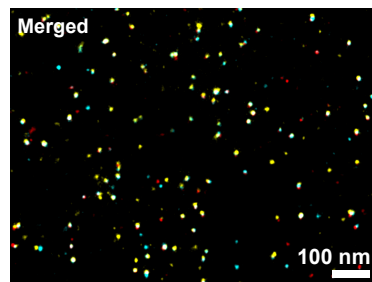**b**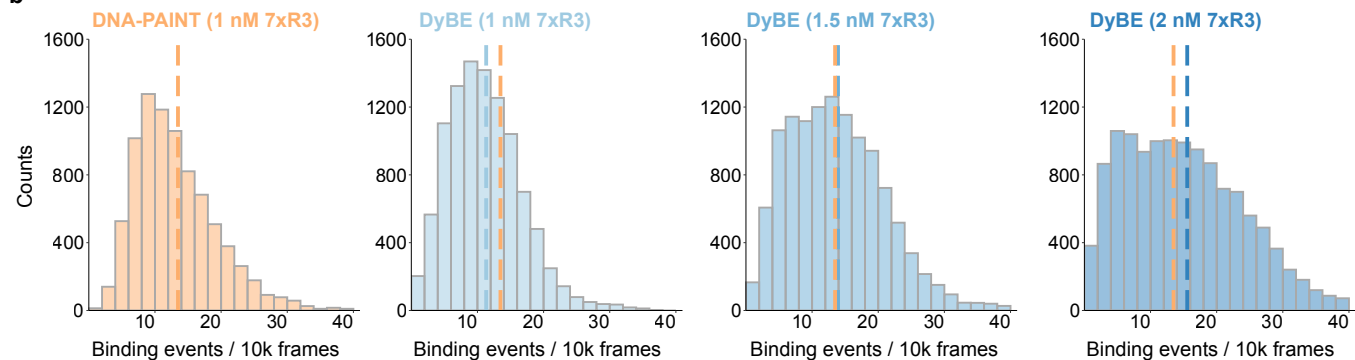

Supplement: Supplementary file 2 — Supporting Information [file ANIE-65-e18685-s002.zip › anie71221-sup-0002-Figures/anie71221-sup-0003-FigureS2.pdf]

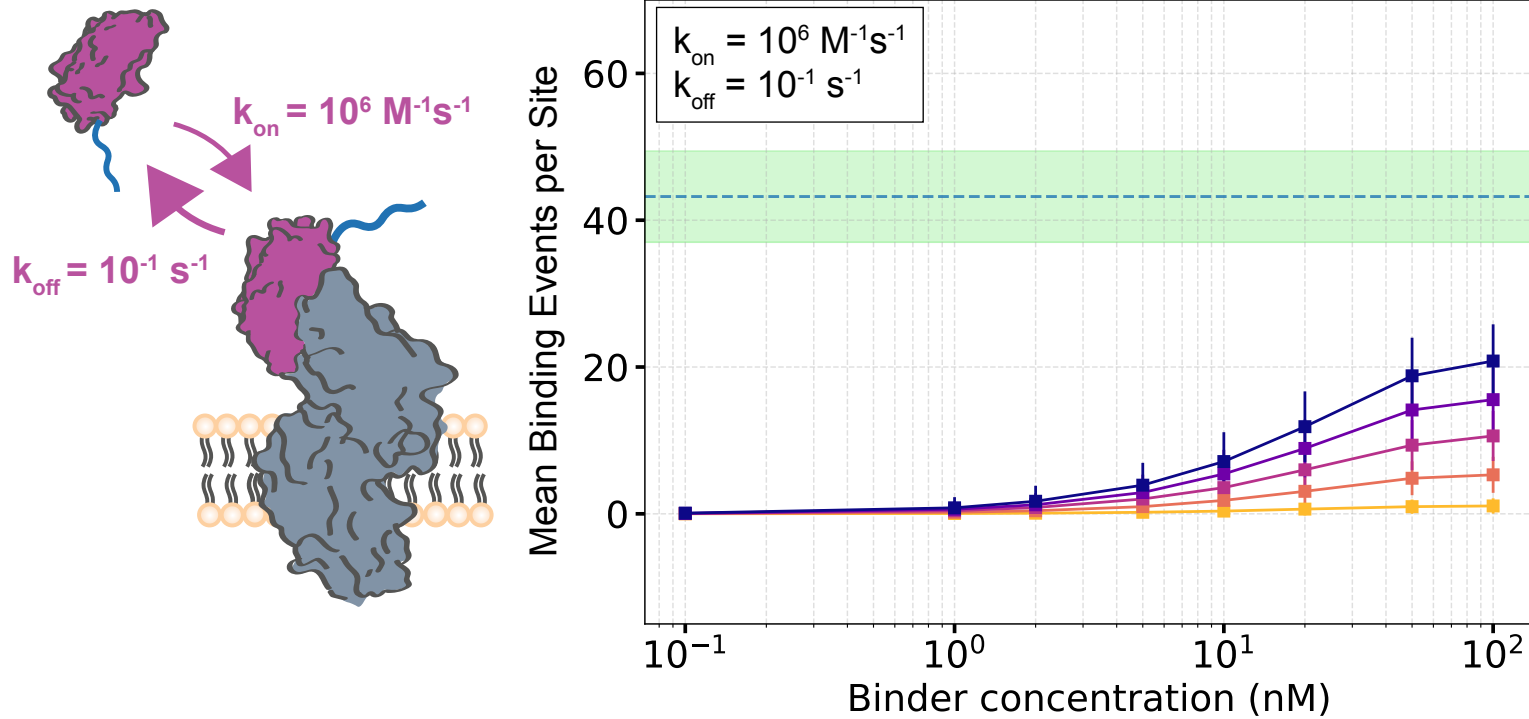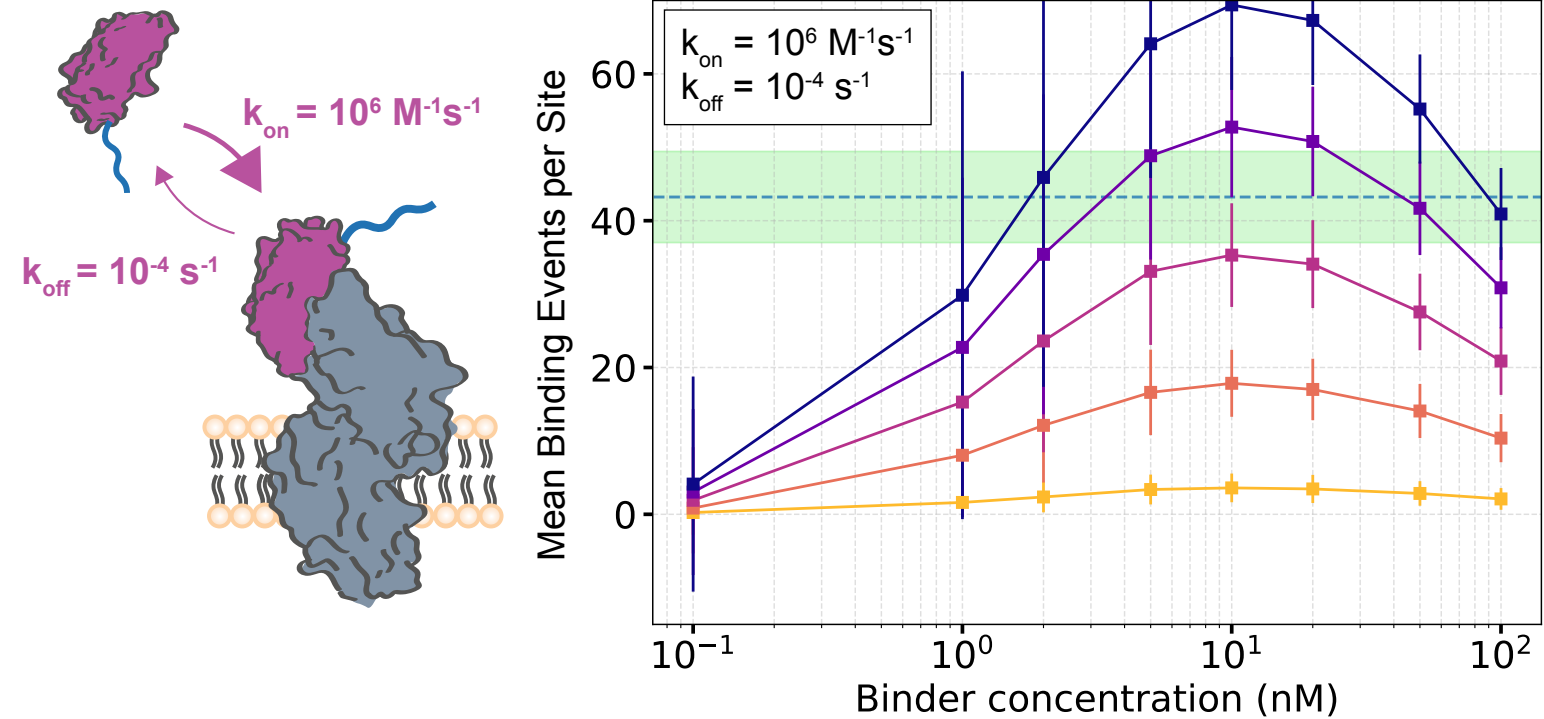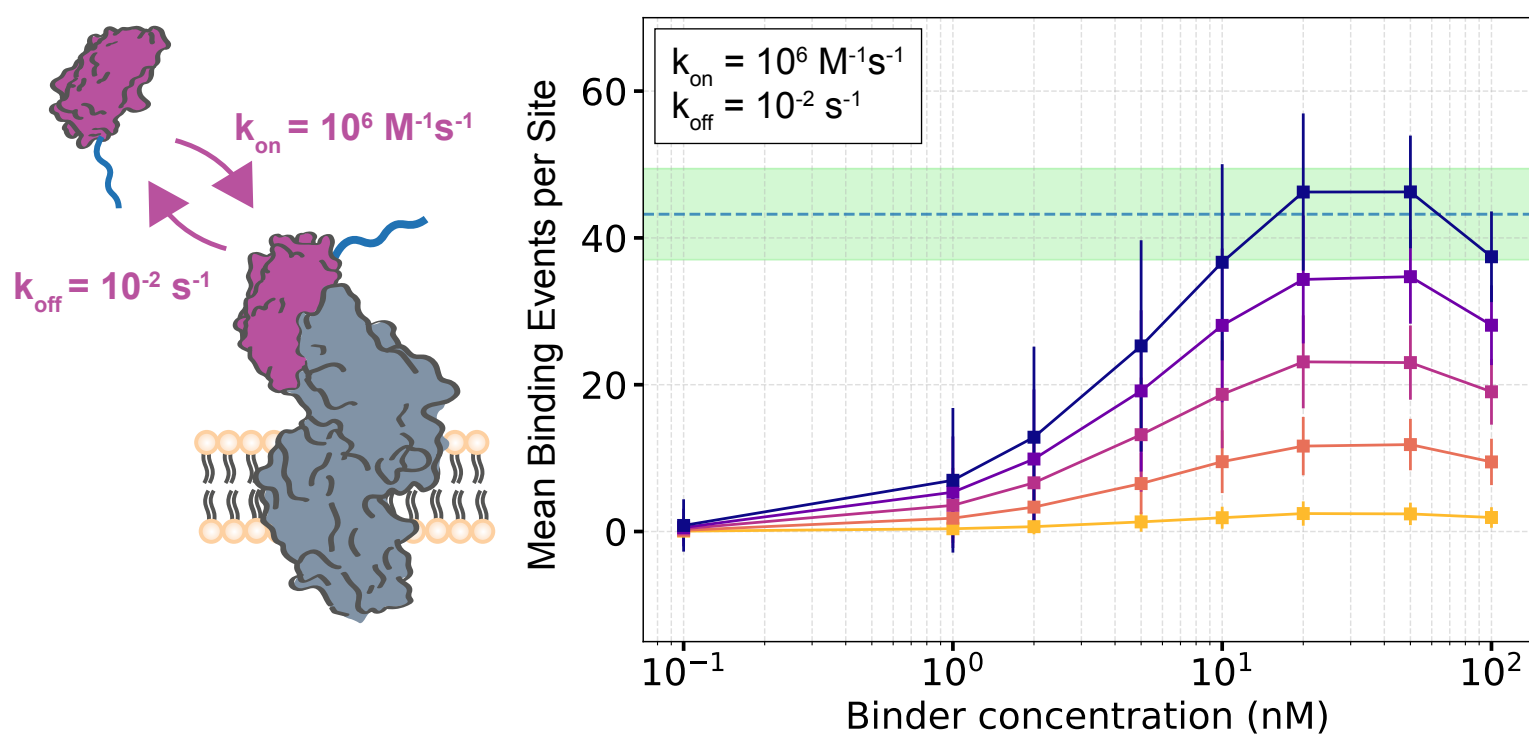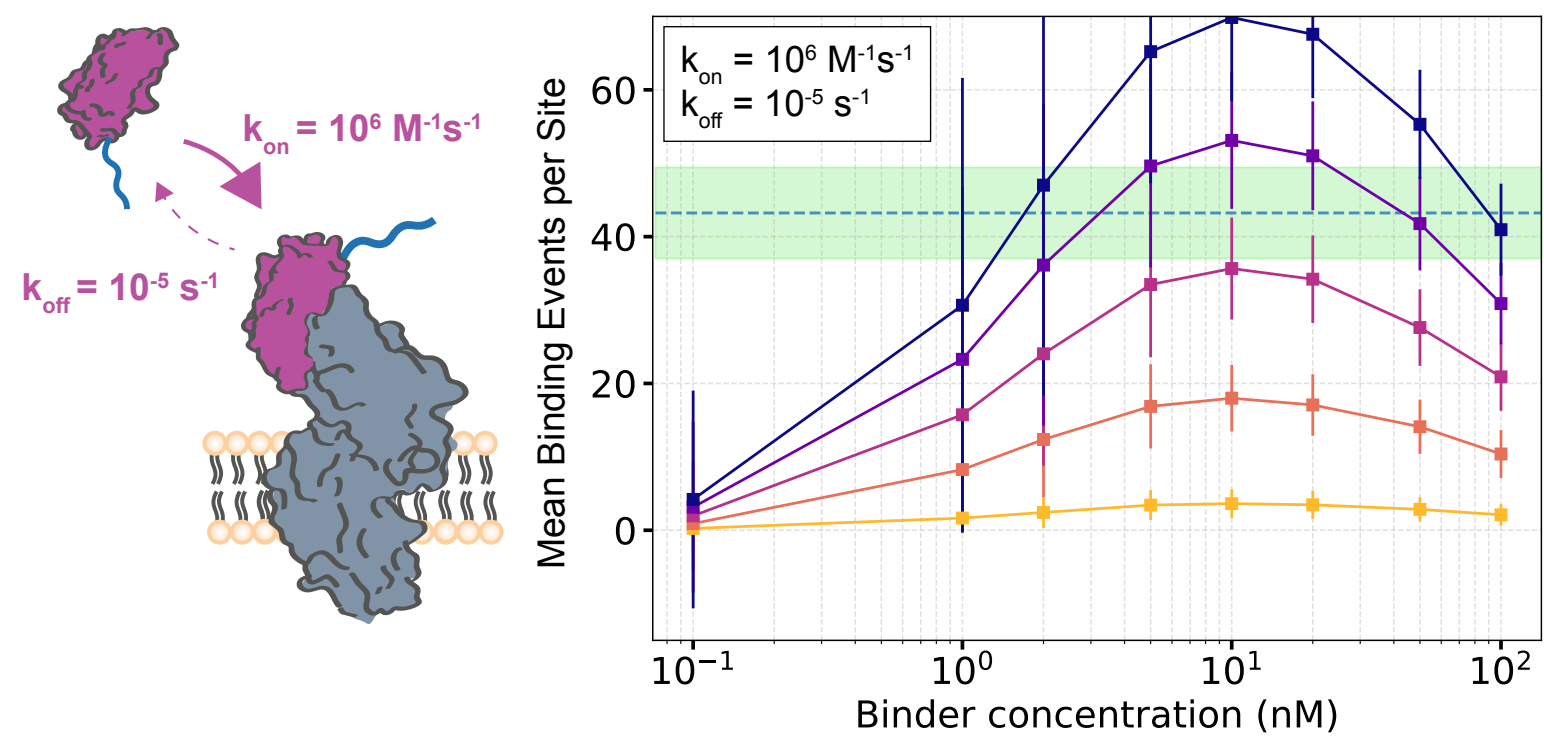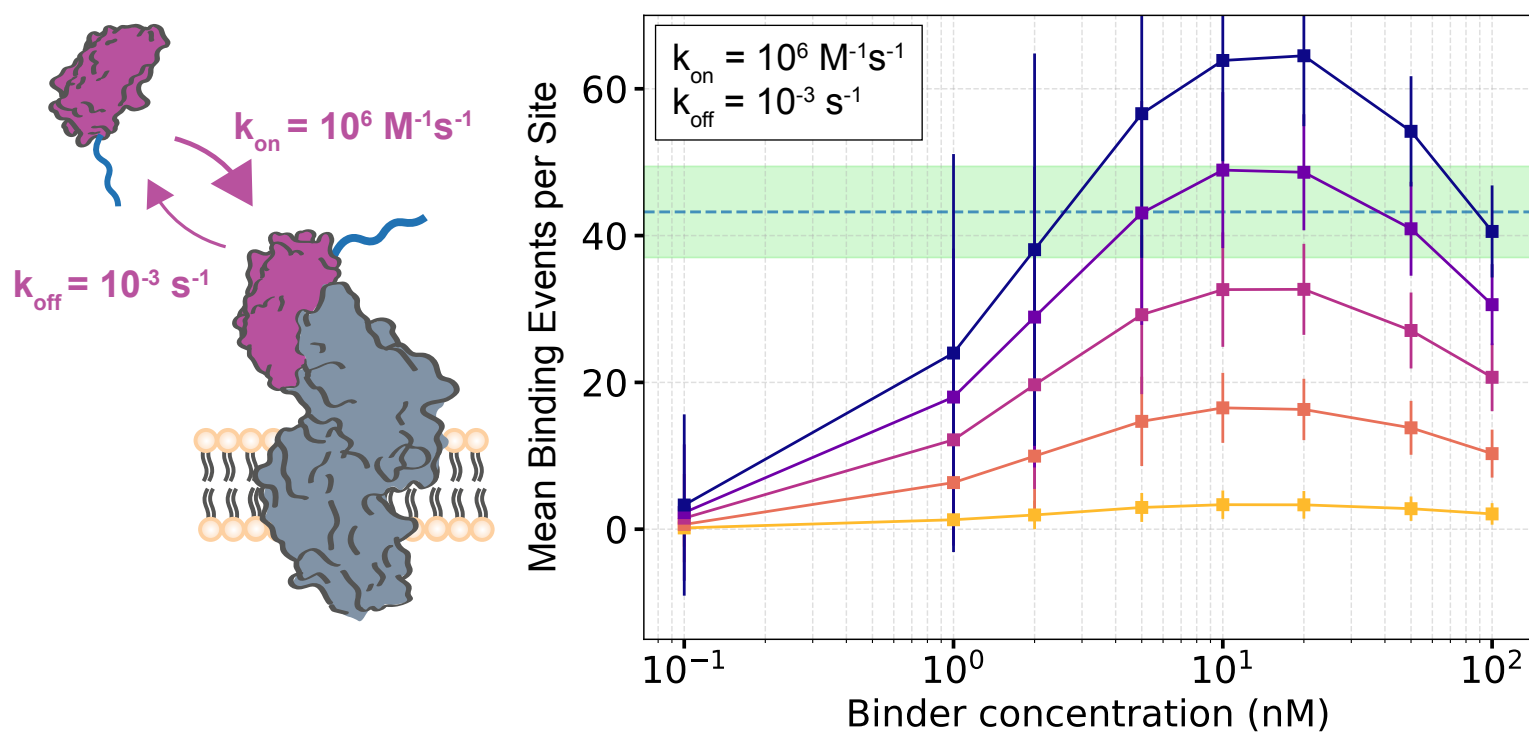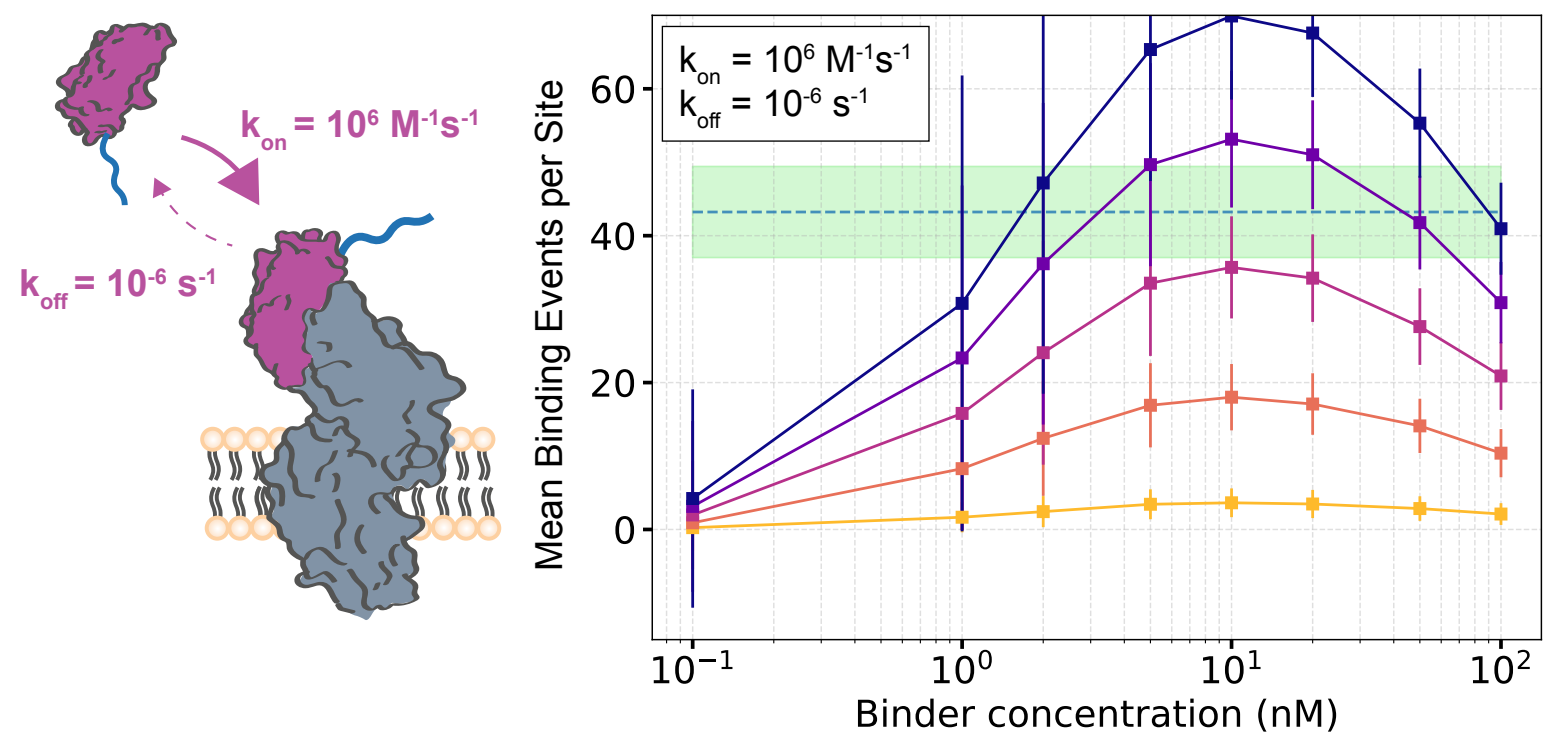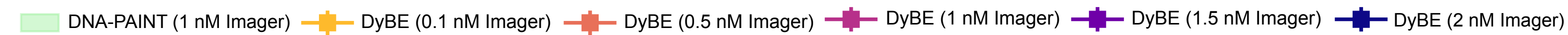

Supplement: Supplementary file 2 — Supporting Information [file ANIE-65-e18685-s002.zip › anie71221-sup-0002-Figures/anie71221-sup-0004-FigureS3.pdf]

**a****DNA-PAINT**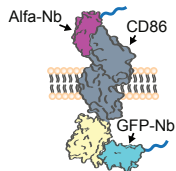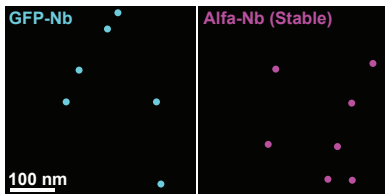**DyBE**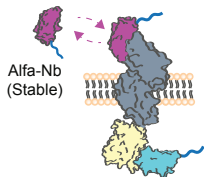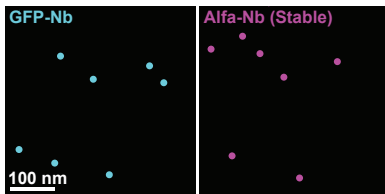**DyBE**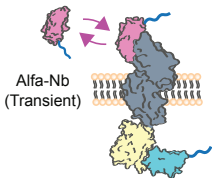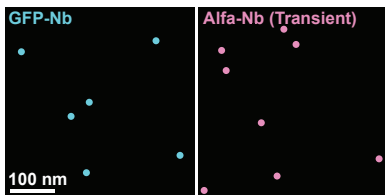**b****DNA-PAINT**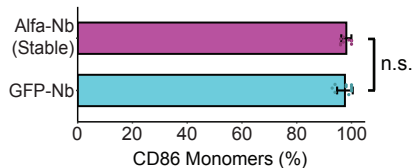**DyBE**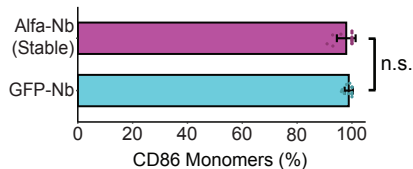**DyBE**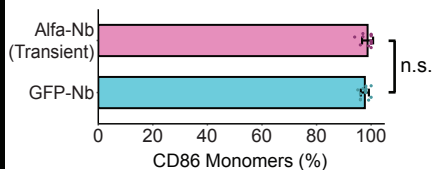

Supplement: Supplementary file 2 — Supporting Information [file ANIE-65-e18685-s002.zip › anie71221-sup-0002-Figures/anie71221-sup-0005-FigureS4.pdf]

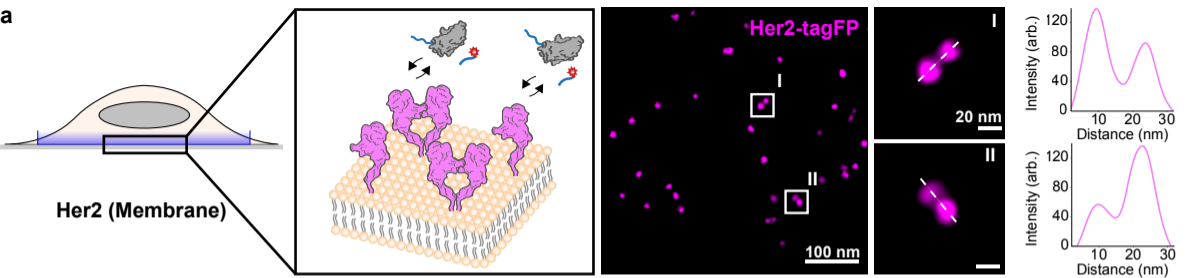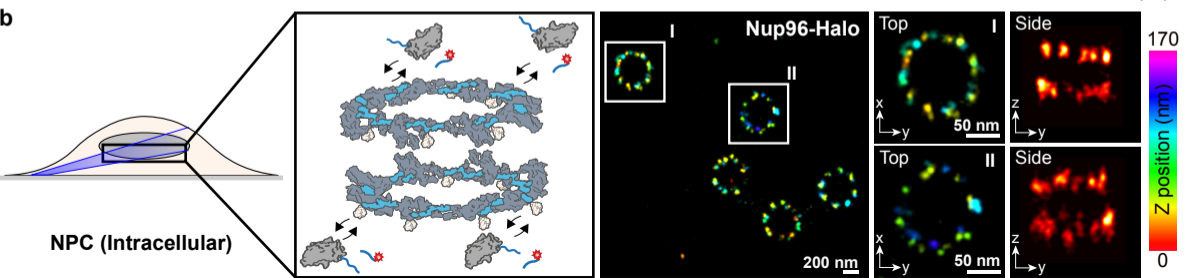

Supplement: Supplementary file 2 — Supporting Information [file ANIE-65-e18685-s002.zip › anie71221-sup-0002-Figures/anie71221-sup-0006-FigureS5.pdf]

**a**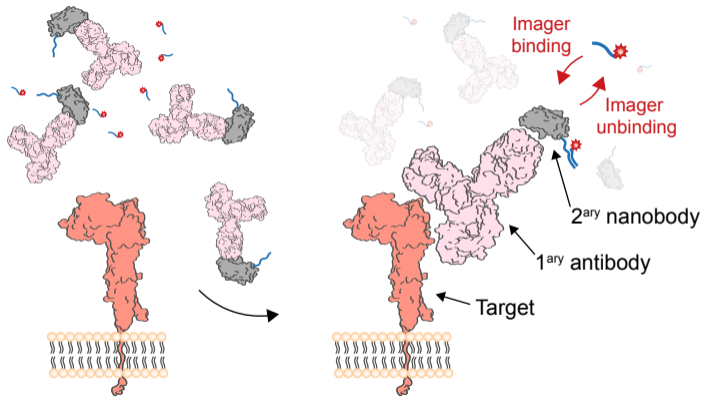**b**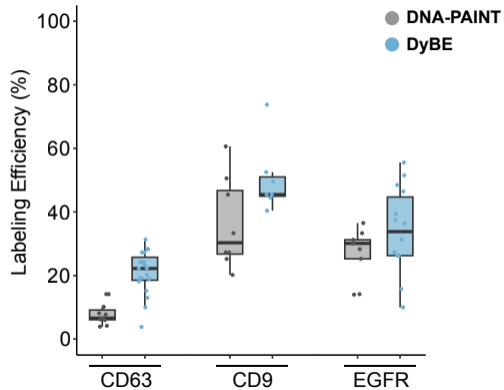

Supplement: Supplementary file 2 — Supporting Information [file ANIE-65-e18685-s002.zip › anie71221-sup-0002-Figures/anie71221-sup-0007-FigureS6.pdf]

**a****Untreated**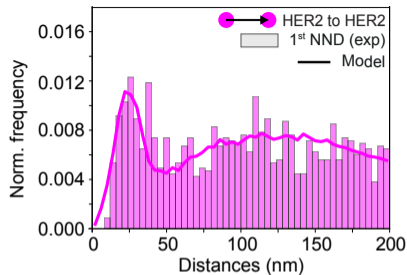**b****EGF-treated**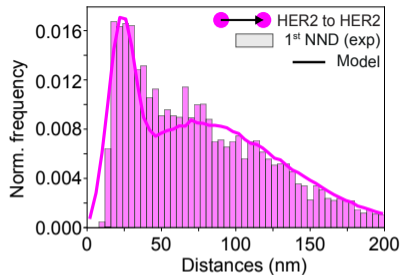**c**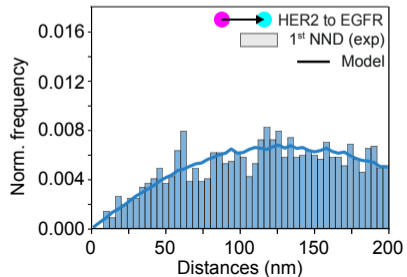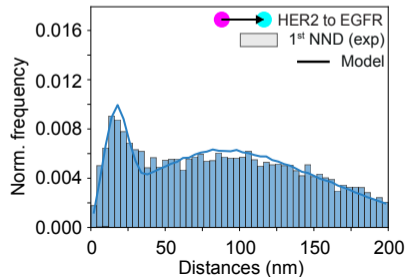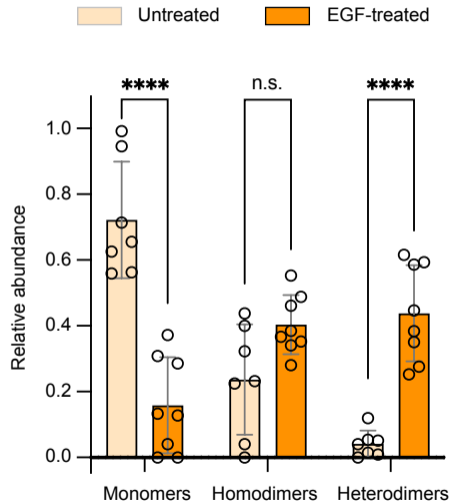

Supplement: Supplementary file 2 — Supporting Information [file ANIE-65-e18685-s002.zip › anie71221-sup-0002-Figures/anie71221-sup-0008-FigureS7.pdf]
